# Perceived discrimination in health care in Germany– results of a population survey

**DOI:** 10.1186/s12939-024-02132-4

**Published:** 2024-02-26

**Authors:** Olaf von dem Knesebeck, Jens Klein

**Affiliations:** Institute of Medical Sociology, University Medical Center Hamburg Eppendorf, Martinistr. 52, 20246 Hamburg, Germany

**Keywords:** Discrimination, Health care, Social inequalities, Online survey, Germany

## Abstract

**Background:**

It has consistently been shown that perceived discrimination is associated with adverse health outcomes. Despite this uncontested relevance, there is a lack of research on the experiences of discrimination in health care. Therefore, the following research questions were addressed: (1) How often do people in Germany report having been discriminated in health care due to different reasons? (2) Which socio-demographic groups are most afflicted by perceived discrimination in health care?

**Methods:**

Analyses are based on a cross-sectional online survey conducted in Germany. An adult population sample was randomly drawn from a panel which was recruited offline (*N* = 2,201). Respondents were asked whether they have ever been discriminated in health care due to the following reasons: age, sex/gender, racism (i.e. migration history, religion, language problems, colour of skin), health issues or disability (i.e. overweight, mental illness/addiction, disability), socio-economic status (SES, i.e. income, education, occupation).

**Results:**

26.6% of the respondents reported discrimination experiences. Perceived discrimination due to health issues or disability was most frequent (15%), followed by age (9%) and SES (8.9%). Discrimination due to racism and sex/gender was less frequently reported (4.1% and 2.5%). Younger age groups, women, and 2nd generation migrants as well as respondents with low income and low education were more likely to report any kind of discrimination in health care. Two groups were found to be at special risk for reporting discrimination in health care across different reasons: women and younger age groups. Discrimination due to racism was more prevalent among respondents who have immigrated themselves than those who were born in Germany but whose parents have immigrated. Discrimination due to SES was significantly associated with (low) income but not with education.

**Conclusions:**

More than a quarter of the adult population in Germany reported experiences of discrimination in health care. Such experiences were more frequent among lower SES groups, migrants, women, and younger people. Results underline the necessity of interventions to reduce the magnitude and consequences of discrimination in health care. Future studies should apply an intersectional approach to consider interactions between social inequality indicators regarding discrimination and to identify risk groups that are potentially afflicted by multiple discrimination.

## Background

According to the stigma concept of Link and Phelan [1] discrimination is the last component of the stigma process that starts with labelling human differences and linking labelled persons to negative stereotypes. Hence, discrimination describes behaviours that act to endorse and reinforce stereotypes, and disadvantage labelled and stigmatized individuals by differential treatment [2]. Discrimination can occur on an interpersonal (i.e. discrimination played out between individuals in everyday life) or a structural level (i.e. discrimination by policies, regulations, and constitutional practice). Human differences that are relevant for stigma are socially selected [1]. In this respect, individuals are potentially discriminated if they have certain characteristics. These characteristics can be related to sex/gender, race/ethnicity, age, socio-economic status (SES), disability or specific diseases [2]. It has consistently been shown that perceived discrimination is associated with multiple adverse health outcomes [3, 4]. Thus, discrimination is an important public health issue that is often discussed as an aspect of health inequities [5–7].

Despite this uncontested relevance, there is a lack of research on the experiences of discrimination in health care [8, 9]. Discrimination in health care can also be structural (e.g. access barriers for patients with a low SES) or interpersonal (e.g. reduced quality of health care communication with low SES patients). The latter is also referred to as provider-based discrimination (i.e. discrimination by occupational groups designated to provide service) [2]. A study examining perceived discrimination in primary health care in 30 European countries found that overall 7% of the respondents felt discriminated in the last 12 months in a primary care practice, with a range between 1.4% and 12.8% in the different countries [10]. In a recent study of Nong et al. [8], 21.4% of the respondents reported that they had experienced discrimination in the U.S. health care system. Racial/ethnic discrimination was the most common type, followed by discrimination based on educational or income level, weight, sex, and age. Women, younger people and lower income groups were more likely to experience discrimination. As it has been shown that perceived discrimination varies between countries and health care systems [10], results yielded in one country cannot be generalized or transferred to another.

In terms of Germany, a recent review revealed that research on discrimination in health care is scarce [9, 11]. The few empirical studies are often limited to regional samples, single aspects of discrimination (e.g. race/ethnicity or age), or specific sectors of health care. In a current study of Bartig et al. [12], it was found that 4% of individuals with a migration history reported (very) frequent discrimination experiences in health or long term care. However, it remained unclear whether in fact the migration history was the reason for perceived discrimination. There are also scattered indications for discrimination in health care among older people and individuals with disability, mental illness, overweight, and a low SES [9], but overall, there is a lack of research in Germany providing an overview.

Against this background, in the present study, the following research questions were addressed: (1) How often do people in Germany report having been discriminated in health care due to different reasons? (2) Which socio-demographic groups are most afflicted by perceived discrimination in health care?

## Methods

### Study design and sample

Analyses are based on a cross-sectional online survey on various experiences in health care. The survey was conducted by a social research institute (forsa) in winter 2022/23. An adult population sample (age 18 + years) was randomly drawn from a panel which was recruited offline via telephone, using a dual-frame approach that included landline as well as mobile phone numbers. The panel is a population-based, representative sample of the adult population living in Germany that is regularly refreshed and currently consists of about 120,000 people. Participants are surveyed regularly on different topics. 5,619 individuals who reported to use the internet were randomly selected from the panel and invited to participate in the present survey via email. After three reminders, *N* = 2,201 individuals participated. Based on a previous study with a similar design [8], we expected about 20–25% of the population to report experiences of discrimination in health care. Therefore, to test differences in perceived discrimination according to the socio-demographic characteristics of the respondents, we aimed at a sample size of about 2,200. Sample was weighted by age (10 groups), sex, federal state, and education (using the iterative proportional fitting approach [13]) according to the official statistics provided by the Federal Statistical Office of Germany [14] and thus adequately represents the adult population in Germany regarding these socio-demographic characteristics. The survey was approved by the Local Psychological Ethics Committee at the Center for Psychosocial Medicine, University Medical Center Hamburg (No. LPEK-0563).

### Measures

Based on previous research [8, 9], perceived discrimination was assessed by asking the respondents whether they have ever been discriminated in health care due to the 12 following reasons: age, sex/gender, migration history, religion, language problems, colour of skin, disability, overweight, mental illness/addiction, income, education, occupation. Respective response options were “yes”, “no”, and “don’t know”. Additionally, a summarized indicator was built showing the number of respondents who experienced at least one of the 12 types of discrimination.

The following socio-demographic factors of the respondents were considered: age, sex, education, income, and migration background. Educational level was assessed using the established CASMIN educational classification which is a hierarchically structured measurement of certificates including the general and vocational qualifications [15]. The nine original CASMIN-levels were merged into four educational groups: low (levels 1a, 1b and 1c), intermediate (2a and 2b), high (2c_gen and 2c_voc), and highest (3a and 3b). Monthly net household income was equalized to consider household size and composition. The variable was further divided into quartiles. Regarding migration background, respondents were categorized into three groups: people who have immigrated themselves (1st generation migrants); people who were born in Germany but whose parents (one or both) have immigrated (2nd generation migrants), and those without a migration background.

### Analyses

Frequencies of perceived discrimination in health care due to the 12 reasons were analysed. In this regard, migration history, religion, language problems, and colour of skin were categorized as “racism” [9]; disability, overweight, and mental illness/addiction were classified as “disability/health issues”, and income, education, and occupation were summarized as “socio-economic status”(SES). Pearson’s chi-square test was applied to test differences in perceived discrimination according to each of the socio-demographic characteristics of the respondents. Finally, multiple logistic regression analyses were conducted with perceived discrimination as dependent variables (categorized reasons and overall discrimination) and the socio-demographic factors being introduced as predictors simultaneously. Odds ratios, 95%-confidence intervals, significances, and explained variances (Nagelkerke’s R^2^) are documented. Statistical procedures were performed with the statistical program package SPSS 27 [16].

## Results

**Table 1:** Sample characteristics (*N* = 2,201)^a^

Characteristic	n (%)
Age (years) *(0)*	
18–40	721 (32.7)
41–59	743 (33.7)
≥ 60	737 (33.5)
Sex *(0)*	
Female	1124 (51.1)
Male	1077 (48.9)
Migration background *(37)*	
No	1670 (77.2)
1st generation	159 (7.4)
2nd generation	335 (15.5)
Income *(326)*	
1st quartile (highest)	471 (25.1)
2nd quartile	466 (24.9)
3rd quartile	481 (25.7)
4th quartile (lowest)	457 (24.3)
Education *(64)*	
Highest	459 (21.5)
High	367 (17.2)
Intermediate	655 (30.6)
Low	656 (30.7)

^a^weighted; number of missing cases in brackets/italics

Distribution of the socio-demographic characteristics (age, sex, migration history, income, and education) in the analysed sample is documented in Table 1.

**Table 2:** Perceived discrimination in health care due to different reasons in Germany (*N* = 2,201)

Perceived discrimination due to…	n (%)
Age	199 (9.0)
Sex/gender	56 (2.5)
Racism Migration history Religion Language problems Colour of skin	89 (4.0) 37 (1.7) 25 (1.1) 48 (2.2) 10 (0.5)
Disability/health issues Disability Overweight Mental illness/addiction	330 (15.0) 47 (2.1) 248 (11.3) 111 (5.0)
Socio-economic status Income Education Occupation	196 (8.9) 156 (7.1) 41 (1.9) 53 (2.4)
At least one type of discrimination	586 (26.6)

Table 2 shows the number of respondents who reported having been discriminated in health care due to the different reasons under study. Perceived discrimination due to age (9%) was more frequent than due to gender or sex (2.5%). Discrimination due to the different aspects of racism ranged between 0.5% (due to colour of skin) and 2.2% (language problems). 4% of the respondents reported at least one of the four aspects. Perceived discrimination due to overweight was most frequent (11.3%). Altogether, 15% of the respondents reported having been discriminated in health care due to health issues or disability. Among the characteristics of the SES, income was most frequently mentioned as a reason for being discriminated (7.1%). More than one quarter (26.6%) of the respondents reported at least one of the 12 types of discrimination.

**Table 3:** Socio-demographic factors of respondents and perceived discrimination in health care (%, *N* = 1,875-2,201)

	Perceived discrimination due to…											
	Age	p*	Sex/ gender	p*	Racism^1^	p*	Socio-economic status^2^	p*	Disability/ health issues^3^	p*	At least one type of discrimination	p*
Age in years 18–40 41–59 60+	14.6 3.2 9.5	< 0.001	5.1 1.7 0.8	< 0.001	6.0 3.8 2.4	0.003	9.4 10.5 6.9	0.046	18.7 17.0 9.2	< 0.001	34.3 27.6 18.2	< 0.001
Sex Male Female	5.2 12.7	< 0.001	0.8 4.2	< 0.001	3.8 4.3	0.581	8.4 9.4	0.376	10.3 19.4	< 0.001	20.8 32.5	< 0.001
Migration background No 1st generation 2nd generation	8.8 4.4 12.6	0.009	2.3 1.3 4.5	0.037	2.6 11.3 7.8	< 0.001	8.7 6.9 10.7	0.329	15.0 11.3 16.1	0.351	25.7 23.3 32.8	0.016
Income 1st quartile 2nd quartile 3rd quartile 4th quartile	7.4 10.1 9.1 9.5	0.529	2.1 2.6 2.9 2.9	0.869	2.5 5.4 3.5 4.4	0.146	6.1 7.3 7.5 14.0	< 0.001	10.4 13.3 17.0 19.3	0.001	21.2 25.7 26.8 33.8	< 0.001
Education Highest High Intermediate Low	9.6 12.0 9.0 7.9	0.186	3.9 6.0 2.0 0.5	< 0.001	4.1 5.4 3.1 4.7	0.262	7.4 9.8 9.0 10.4	0.393	11.5 11.5 15.4 19.6	< 0.001	24.6 27.8 26.4 29.5	0.307

* Significance of Chi^2^-Test; ^1^migration, religion, language problems, skin colour; ^2^income, education, occupation; ^3^disability, overweight, mental illness, addiction

Age-related discrimination was more often reported by young respondents (18 to 40 years), females, and 2nd generation migrants (Table 3). Younger people, females and respondents with high education more often perceived discrimination due to gender or sex. Perceived racist discrimination (including migration, religion, language problems, and skin colour) was more pronounced among younger respondents and those with a migration history. People who were younger than 60 years and individuals with low income (4th quartile) felt more often discriminated due to their SES (according to income, education, and occupation). Perceived discrimination due to health issues or disability (disability, overweight, mental illness, and addiction) was significantly associated with lower age, female sex, lower income, and lower education. Finally, the rate of those who reported at least one type of discrimination was increased among younger people, females, 2nd generation migrants, and respondents with a low income.

**Table 4:** Associations between socio-demographic factors of respondents and perceived discrimination in health care: Odds ratios, (confidence intervals), and significances (*N* = 1,840)

	Perceived discrimination due to…					
	Age	Sex/gender	Racism^1^	Socio-economic status^2^	Disability/health issues^3^	At least one type of discrimination
Age in years 18–40 41–59 60+	1.00 0.16 (0.09–0.28)*** 0.55 (0.36–0.82)**	1.00 0.52 (0.26–1.06) 0.17 (0.06–0.51)**	1.00 0.54 (0.30–0.99)* 0.30 (0.15–0.61)**	1.00 1.21 (0.80–1.83) 0.72 (0.45–1.15)	1.00 0.62 (0.44–0.87)** 0.27 (0.19–0.40)***	1.00 0.64 (0.49–0.84)** 0.34 (0.25–0.47)***
Sex Male Female	1.00 2.59 (1.83–3.67)***	1.00 7.30 (3.22–16.53)***	1.00 1.16 (0.72–1.88)	1.00 1.24 (0.89–1.72)	1.00 2.30 (1.75–3.02)***	1.00 2.07 (1.67–2.57)***
Migration background No 1st generation 2nd generation	1.00 0.52 (0.23–1.20) 1.27 (0.84–1.94)	1.00 0.57 (0.12–2.64) 1.19 (0.58–2.44)	1.00 5.44 (2.80-10.54)*** 2.78 (1.58–4.87)***	1.00 0.86 (0.42–1.73) 1.17 (0.76–1.81)	1.00 0.69 (0.37–1.27) 1.13 (0.79–1.61)	1.00 0.93 (0.60–1.44) 1.38 (1.04–1.83)*
Income 1st quartile 2nd quartile 3rd quartile 4th quartile	1.00 1.42 (0.88–2.30) 1.14 (0.70–1.86) 1.10 (0.67–1.80)	1.00 1.48 (0.60–3.61) 1.71 (0.72–4.08) 1.31 (0.54–3.21)	1.00 2.35 (1.13–4.87)* 1.45 (0.67–3.16) 1.47 (0.68–3.17)	1.00 1.15 (0.68–1.93) 1.19 (0.70-2.00) 2.28 (1.41–3.67)***	1.00 1.24 (0.82–1.87) 1.51 (1.01–2.25)* 1.56 (1.05–2.33)*	1.00 1.24 (0.90–1.70) 1.23 (0.89–1.68) 1.56 (1.14–2.13)**
Education Highest High Intermediate Low	1.00 1.35 (0.82–2.21) 1.03 (0.63–1.66) 1.29 (0.76–2.19)	1.00 1.40 (0.68–2.89) 0.56 (0.24–1.27) 0.22 (0.06–0.77)*	1.00 1.17 (0.57–2.42) 1.02 (0.49–2.12) 1.69 (0.81–3.55)	1.00 1.14 (0.68–1.93) 1.10 (0.67–1.82) 1.36 (0.81–2.29)	1.00 0.92 (0.58–1.48) 1.49 (0.99–2.25) 2.70 (1.76–4.14)***	1.00 1.12 (0.79–1.59) 1.26 (0.91–1.73) 1.88 (1.34–2.64)***
Nagelkerke’s R^2^	0.114	0.182	0.092	0.036	0.107	0.094

* *p* < 0.05, ** *p* < 0.01, *** *p* < 0.001; ^1^migration, religion, language problems, skin colour; ^2^income, education, occupation; ^3^disability, overweight, mental illness, addiction

Table 4 shows the results of the multiple logistic regression analyses with the socio-demographic predictors being introduced as predictors simultaneously. In terms of age-related discrimination, the two older age groups were significantly less likely to report discrimination compared to the youngest group (18 to 40 years) after adjustment for all other socio-demographic factors. Moreover, women were about 2.6 times more likely to perceive discrimination due to age than men. Regarding perceived sexist discrimination in health care, people aged 60 years and older as well as people having a low education were significantly less likely to report this. Female respondents had a more than 7 times increased likelihood compared to males. Probability of perceiving racist discrimination was increased among 1st (odds ratio 5.44) and 2nd generation migrants (odds ratio 2.78) (compared to people without migration background) as well as respondents belonging to the 2nd highest income quartile (odds ratio 2.35) while it was significantly decreased among the two higher age groups (odds ratio 0.30 and 0.54). SES-related discrimination was significantly increased in the lowest income group in comparison to the highest income group. The two older age groups were significantly less likely to report discrimination due to health issues or disability compared to the youngest group, while females were more likely. Likelihood of perceived discrimination in this regard was increased among lower income and education groups. The summarized indicator of perceived discrimination was significantly associated with all socio-demographic characteristics of the respondents showing strongest relation with age and sex. Explained variance (Nagelkerke’s R^2^) ranged between 4% and 18% depending on the indicator of perceived discrimination.

## Discussion

### Summary and interpretation

In the present study, two research questions were addressed: (1) How often do people in Germany report having been discriminated in health care due to different reasons (age, sex/gender, racism (i.e. migration history, religion, language problems, colour of skin), disability or health issues (i.e. disability, overweight, mental illness/addiction), and SES (i.e. income, education, occupation))? (2) Which socio-demographic groups are most afflicted by perceived discrimination in health care? In terms of the first question, results showed that 26.6% of the respondents reported having experienced at least one type of discrimination under study. Perceived discrimination due to disability or health issues was most frequent (15%), followed by age (9%) and SES (8.9%). Among the health issues, discrimination due to overweight was most frequent (11.3%). This result is supported by studies indicating weight bias in health care [17]. Discrimination due to racism and sex/gender was less frequently reported (4.1% and 2.5%). As to the second question, younger age groups, women, and 2nd generation migrants as well as respondents with low income and low education were more likely to report any kind of discrimination in health care. Two groups were found to be at special risk for reporting discrimination in health care across different reasons: women and younger age groups. Discrimination due to racism was more prevalent among respondents who have immigrated themselves than those who were born in Germany but whose parents have immigrated. Discrimination due to SES was significantly associated with (low) income but not with education.

To the best of our knowledge, this is one of the first studies providing an overview of different types of perceived discrimination in health care in Germany. Prevalence of perceived discrimination presented here is higher than in a U.S. study by Nong et al. [8] that found 21.4% of the respondents reporting any experiences of discrimination in health care. In contrast to our findings, racial/ethnic discrimination was the most common type in the U.S., followed by discrimination based on educational or income level, weight, sex, and age. Similar to our study, women, younger people and lower income groups were more likely to experience discrimination in the U.S. health care system. A European study focussing on discrimination in primary care [10] also found that lower income groups and younger people tend to feel more discriminated while sex differences were inconsistent between the European countries. The overall consistent association with age may indicate that younger people have a stronger feeling for discrimination.

Several studies have shown that perceived discrimination affects mental and physical health through physiologic, psychosocial and behavioural factors [3, 4]. Moreover, experiences of discrimination in health care are expected to act as a barrier increasing the likelihood of forgone care [18, 19] and lower utilization of health care [20, 21]. Nong et al. [22] found that such experiences also are associated with lower trust in the health care provider and a reduced willingness to share information with the provider, possibly resulting in lower quality of medical decision-making and care. These findings, together with the results presented here underline that discrimination is an important aspect of inequalities in health and health care [5–7, 23, 24].

### Limitations

Some limitations have to be considered when interpreting our results. Analyses were based on an online survey. Although a random sample was drawn from a panel which was recruited offline, only those who use the internet were included. It can be expected that, for example very old people or individuals with severe health problems and limitations may be underrepresented in this group. Moreover, as only about 39.2% of the invited persons participated, a selection bias cannot be ruled out. To reduce this potential bias, data was weighted by age, sex, federal state, and education according to the official statistics using an iterative proportional fitting approach [12]. Furthermore, analyses were restricted to individuals who were able to read German. This has to be especially kept in mind, when interpreting results on discrimination due to language problems. In terms of measures, self-reports of discrimination experiences were used. Such self-reports can be biased as respondents may perceive or report less or more discrimination than actually exists [25, 26]. Moreover, our discrimination measure does not differentiate between structural and interpersonal discrimination. Furthermore, the measure in a way is crude because we asked whether the respondent have *ever* been discriminated in health care and thus, did not specify a time frame. As for discrimination due to disability and health issues, only three indicators were included (disability, overweight, and mental illness/addiction), i.e. many conditions were not addressed. In terms of racism, discrimination due to religion was included although it is less apparent than for example colour of skin. Generally, the studied reasons for discrimination vary regarding their visibility. Finally, the study was conducted in Germany and results cannot be transferred to other countries.

## Conclusions

More than a quarter of the adult population in Germany reported experiences of discrimination in health care. Such experiences were more frequent among lower SES groups, migrants, women, and younger people. Our results underline the necessity of public health and health care interventions to reduce the magnitude and consequences of discrimination in health care. Future studies should apply an intersectional approach [27] to consider interactions between social inequality indicators regarding discrimination and to identify risk groups that are potentially afflicted by multiple discrimination.

## Data Availability

No datasets were generated or analysed during the current study.

## Abbreviations

SES: socio-economic status
